# P-1500. Preclinical Data Supporting Development of a Novel CMV Vaccine

**DOI:** 10.1093/ofid/ofaf695.1684

**Published:** 2026-01-11

**Authors:** Sumi Biswas, Prakash Bhuyan, Sophie Porret, Antonia Hook, David Bitto, Jeanette Wagener, Lesley A H Bowman

**Affiliations:** SpyBiotech, Oxford, England, United Kingdom; SpyBiotech, Oxford, England, United Kingdom; SpyBiotech, Oxford, England, United Kingdom; SpyBiotech, Oxford, England, United Kingdom; SpyBiotech, Oxford, England, United Kingdom; SpyBiotech, Oxford, England, United Kingdom; SpyBiotech, Oxford, England, United Kingdom

## Abstract

**Background:**

Human cytomegalovirus causes significant and permanent disabilities in children. In the US, it is estimated that 16000 newborns are born with CMV infections every year which can lead to deafness, blindness, still birth and developmental delays. Unfortunately, there are no antiviral treatments, which makes the need for a preventative vaccine even more significant. We present the supportive preclinical data for a virus like particle (VLP) vaccination approach.

**Methods:**

The vaccine is a Hepatitis B surface antigen VLP decorated with the CMV pentameric complex (gH, gL, UL128, UL130, and UL131A) using a proprietary s.pyogenes protein. Pre-clinical studies have been performed in BALB/c mice to compare the immunogenicity and neutralising potential of HBsAg VLP displaying the pentamer (1 μg and 0.1 μg) versus pentamer protein alone (1 μg and 0.1 μg) in the presence and absence of adjuvants.

**Results:**

The unadjuvanted pentamer-HBsAg VLP was 20 to 30 times more immunogenic than the same dose of unadjuvanted pentamer protein (post-prime: 168 AU vs 7 AU; post-boost: 3400 AU vs 117 AU), and had substantially more neutralising potential in epithelial cells (post-prime: NT50 = 22 vs 0; post-boost: NT50 = 3036 vs 4; post- boost-boost: NT50 = 5672 vs 993) and fibroblasts (post-boost: NT50 = 43 vs 0; post- boost-boost: NT50 = 93 vs 26). Post-vaccination virus neutralization potency remained stable through the end of study supporting that the VLP approach can be durable.

**Conclusion:**

These preclinical data support further development of this CMV vaccine candidate. Pending supportive safety, immunogenicity and durability data from an ongoing Phase 1 trial, the interruption of CMV infection can be potentially achieved in toddlers, adolescents and young women of childbearing potential to maximise the impact of such a vaccine.

**Disclosures:**

All Authors: No reported disclosures

